# Comparative Pharmacokinetics and Egg Residues of Amoxicillin, Single and in Combination with Bromhexine, in Laying Hens

**DOI:** 10.3390/pathogens13110982

**Published:** 2024-11-08

**Authors:** Jeong-Won Kim, Ji-Soo Jeong, Jin-Hwa Kim, Chang-Yeop Kim, Eun-Hye Chung, So-Young Boo, Soo-Ha Lee, Je-Won Ko, Tae-Won Kim

**Affiliations:** 1 College of Veterinary Medicine (BK21 FOUR Program), Chungnam National University, 99 Daehak-ro, Daejeon 34131, Republic of Korea; lilflflb@gmail.com (J.-W.K.); jisooj9543@gmail.com (J.-S.J.); jinhwa926@g.cnu.ac.kr (J.-H.K.); 963ckdduq@gmail.com (C.-Y.K.); ksissb1293@gmail.com (E.-H.C.); labong1966@gmail.com (S.-Y.B.); suhai2729@gmail.com (S.-H.L.); 2 Division of Radiation Biomedical Research, Korea Institute of Radiological and Medical Science, 75 Nowon-ro, Nowon-gu, Seoul 01812, Republic of Korea

**Keywords:** amoxicillin, bromhexine, egg residue, laying hens, pharmacokinetics

## Abstract

The need for antibiotics in commercial laying hens is increasing owing to intensive farming systems. Amoxicillin trihydrate (AMX), an aminopenicillin β-lactam antibiotic, exerts broad bactericidal activity. However, its short half-life necessitates frequent administration to ensure efficacy, thus limiting its use. Herein, we investigated the effect of concurrent administration of bromhexine hydrochloride (BRM), a mucolytic agent, on AMX pharmacokinetics, performing a comparative pharmacokinetic analysis of AMX administration alone and in combination with BRM. AMX (50 mg/kg) was administered by oral gavage once daily for three days alone or in combination with 10 mg/kg BRM. Plasma and egg samples were collected to evaluate pharmacokinetic profiles and egg residues. The area under the curve and maximum plasma concentration values were significantly higher in the AMX + BRM group than the AMX only group. However, there were no significant differences in AMX half-life in the elimination phase (T_1/2_), elimination rate constant (k_el_), or apparent clearance (CL/F) values. In the egg residue study, the withdrawal period for AMX was 5 days in both groups, with no significant difference when using the maximum residue limit (MRL) of 10 μg/kg. The concentration of BRM in the eggs remained at 100 μg/kg up to the fourth day following drug administration. Conclusion: These results confirmed that BRM co-administration increased systemic exposure to AMX, with a negligible residual impact of amoxicillin in eggs.

## 1. Introduction

Intensive egg-hen farming systems provide cost-effective protein sources but leave laying hens susceptible to upper respiratory diseases caused by bacterial or viral infections, thus increasing the need for antibiotics [1]. The use of antibiotics in laying hens not only enhances animal health, but also improves the efficiency of egg production, thus boosting economic benefits [2]. However, the indiscriminate use of antibiotics in livestock products, which often rely on their effectiveness, can lead to issues such as drug residues, which pose serious health risks to consumers [3]. As such, antibiotics must only be used in compliance with withdrawal periods that meet the maximum residue limits (MRLs) for the relevant drugs [4]. Therefore, various studies are needed to determine the withdrawal period that meets the MRL.

Amoxicillin trihydrate (AMX) is an aminopenicillin β-lactam antibiotic drug which exerts broad bactericidal activity by binding to proteins essential for bacterial cell wall synthesis, resulting in cell death [5,6]. Owing to its broad-spectrum antibacterial effect and low cost, AMX is widely applied in farm animals, including laying hens, to prevent and treat bacterial infections; however, caution must be exercised regarding residue issues [7]. Residues may vary according to the experimental conditions; however, AMX has been reported to have a relatively short elimination half-life of approximately 1 h in chickens [5,8,9]. Previously, Abo El-Sooud et al. [5] reported that the administration of 10 mg/kg of oral amoxicillin at 24 h intervals, which is the generally recommended dose, was not sufficient to treat systemic infections in poultry. Because of the need for frequent administration to maintain drug efficacy, various alternative methods are being researched to reduce administration frequency and enhance drug efficacy [10].

Bromhexin hydrochloride (BRM) is a synthetic derivative of vasicin, an active ingredient in *Adhatoda vasica*, and induces natural coughing and mucus normalization [11]. BRM, used as a mucolytic agent for treating respiratory diseases, has shown in clinical trials that the co-administration of AMX and BRM significantly reduced symptom scores from lower respiratory tract infections compared to AMX alone [12,13]. BRM has been reported to clear mucus and reduce its viscosity, thereby increasing the concentration of co-administered antimicrobials in the respiratory tract, which can facilitate the distribution and penetration of antibiotics within pulmonary tissues [14]. Additionally, previous studies have reported that BRM increases the concentration of AMX in lung tissue, which is expected to enhance its efficacy against lung infections [15,16].

While there are many studies related to the efficacy of drug combinations involving BRM, research on the impact of BRM on the pharmacokinetics of co-administered antibiotics, such as AMX, that could assess the extent of systemic exposure, is lacking. Moreover, although increased absorption of AMX in laying hens could be advantageous from a therapeutic perspective, concerns regarding potential residues in eggs need to be explored. In the present study, we explored the pharmacokinetics of AMX following administration of AMX alone or in combination with BRM in laying hens to determine the effect of BRM on the pharmacokinetics of AMX and its potential impact on AMX residues in eggs.

## 2. Methods

### 2.1. Chemicals and Reagents

Analytical standards of AMX trihydrate (PHR1127, purity >95%) and BRM hydrochloride (17343, purity >98%) were obtained from Sigma–Aldrich (St. Louis, MO, USA). Other chemicals, including acetonitrile, ammonium acetate, dichloromethane, trichloroacetic acid, and methanol, were purchased from Sigma–Aldrich.

### 2.2. Animal Experiments

Twelve laying hens (Hy-line Brown, 2.4 ± 0.3 kg,) were obtained from Sungwonfarm (Sejong, Republic of Korea), and acclimatized in cages for one week. Food and tap water were provided daily and ad libitum, respectively. Twelve hens were randomly assigned to the AMX and AMX + BRM groups (n = 6 per group). The AMX group was administered 50 mg/kg AMX analytical standard via oral gavage, while the AMX + BRM group was administered 50 mg/kg AMX and 10 mg/kg BRM analytical standards via oral gavage.

The drugs were completely dissolved in tap water and administered daily for 3 days. In both groups, the blood (1 mL) was collected in heparinized tubes at 0, 0.25, 0.75, 1, 3, 6, 8, 10, 12, 24, 36, 48, 72, and 96 h after the last administration. The blood was collected from the wing vein. For egg residue studies, eggs were collected up to four days following the final drug administration. Plasma and eggs were frozen at −70 °C until drug analysis. All experiments were approved by the Institutional Animal Care and Use Committee of Chungnam National University (Daejeon, Republic of Korea; 202206A-CNU-086), and were performed in compliance with the Guidelines for Animal Experiments of Chungnam National University.

### 2.3. Sample Preparation

For AMX, eggs (5 g) were homogenized in 10 mL acetonitrile for 10 min, and sonicated for 15 min. The homogenized mixture was centrifuged at 2500× *g* for 10 min using a centrifuge (Allsheng, Hangzhou, China) and the supernatant was collected in another tube. This process was repeated twice to obtain 20 mL supernatant, then the supernatant was mixed with 1 mL of ammonium acetate (3.89 M, pH 6.74) and 10 mL of dichloromethane. The mixture was centrifuged at 2500× *g* for 10 min. The supernatant (1 mL) was evaporated with nitrogen at 50 °C using a nitrogen evaporator (Synergene, Daejeon, Republic of Korea), and suspended in 0.5 mL of 2% acetonitrile. The solution was subsequently filtered through a 0.22 µm membrane filter (Sigma-Aldrich, St. Louis, MO, USA), and injected into the ultra-performance liquid chromatography-tandem mass spectrometry (UPLC-MS/MS) equipment for analysis. Plasma samples were processed at 1/10 volume using the above protocol.

For BRM, eggs (5 g) were homogenized in 20% trichloroacetic acid (0.5 mL) and acetonitrile (20 mL) for 10 min, and centrifuged at 2500× *g* for 10 min. The supernatant was then adsorbed using an activated HLB cartridge (Waters, Milford, MA, USA), and eluted with 5 mL of a methanol:acetonitrile (1:1, *v*/*v*) solution. The eluted solution was then filtered through a 0.22 µm membrane filter and injected into the UPLC-MS/MS for the analysis. Plasma samples were processed at 1/10 volume using the above protocol.

### 2.4. UPLC-MS/MS Analysis and Method Validation

A UPLC-MS/MS system (UPLC 1290 TQ 6470, Agilent Technologies, Palo Alto, CA, USA) equipped with an Eclipse Plus C18 column (2.1 × 100 mm, 3.5 μm; Agilent Technologies), delivering a flow rate of 0.3 mL/min, was used for chromatography analysis. Distilled water was obtained using a Milli-Q IQ 7000 ultrapure water purification system (Sigma-Aldrich). The mobile phase comprised 0.1% formic acid in distilled water (A) and 0.1% formic acid in acetonitrile (B). A gradient solvent program was run with the following conditions: 0 min (90% A), 0–3 min (90–10% A), and 3–4 min (10–90% A). The methods used to analyze the plasma and eggs were validated. AMX and BRM were detected in product ion scan mode. The collision energies of AMX and BRM were determined to be 40 eV and 20 eV, respectively. For quantitative analysis, data acquisition was conducted in the multiple-reaction-monitoring (MRM) mode, monitoring transition m/z 366.1→134 (for AMX), m/z 375→114.1 (for BRM) in a positive ion mode. The sample injection volumes for AMX and BRM were 5 and 10 µL, respectively. The limits of detection (LOD) and quantification (LOQ) were established, according to the International Conference on Harmonization (ICH) guidelines using a signal-to-noise (S/N) ratio of 10. The intraday precision was assessed by analyzing the quality control standards three times per day (10, 100, and 1000 ng/mL of AMX and 10, 50, and 100 ng/mL of BRM). Inter-day precision was evaluated by analyzing the quality control standards over three consecutive days (10, 100, and 1000 ng/mL of AMX and 10, 50, and 100 ng/mL of BRM). The recoveries of AMX and BRM were calculated by comparing the theoretical concentrations of AMX and BRM, spiked into the blank plasma and eggs with the detected concentrations.

### 2.5. PK and Statistical Analysis

Plasma concentrations were determined by noncompartmental analysis (NCA) using PKanalix2024R1 (Lixoft, Antony, France).

The following PK parameters of AMX and BRM were calculated: maximum concentration (C_max_), time required to reach C_max_ (T_max_), area under the curve (AUC), apparent clearance (CL/F), half-life in the elimination phase (T_1/2_), elimination rate constant (k_el_), and apparent volume of distribution (V_d_/F). To set the withdrawal period for AMX and BRM, log-linear regression with a 99% upper tolerance limit and 95% confidence interval was applied to the egg concentration.

Statistical analyses were performed by analyzing the pharmacokinetic parameters using the Shapiro–Wilk test (SPSS software ver. 21.0; IBM Corp., Armonk, NY, USA) to assess normality. The concentrations of AMX and BRM were calculated using WT 1.4 software, adopted by the Committee for Veterinary Medicinal Products of European Medicines Agency (EMA). To assess statistical significance, an unpaired *t*-test was performed, and the significance was marked as *p* < 0.05 or *p* < 0.01.

## 3. Results

### 3.1. Pharmacokinetic Study

The UPLC-MS/MS method was validated, with results showing specificity and linearity (0–10 µg/mL) for AMX and BRM in the plasma (R^2^ > 0.99). The LOD and LOQ of AMX were 2.346 and 7.743 ng/mL, respectively, whereas those of BRM were 1.440 and 4.750 ng/mL, respectively. The recovery of AMX and BRM for plasma was 95.2 ± 3.7% and 92.4 ± 4.9%, respectively. All intra- and inter-day relative standard deviations for the plasma were < 7%.

**Figure 1:**
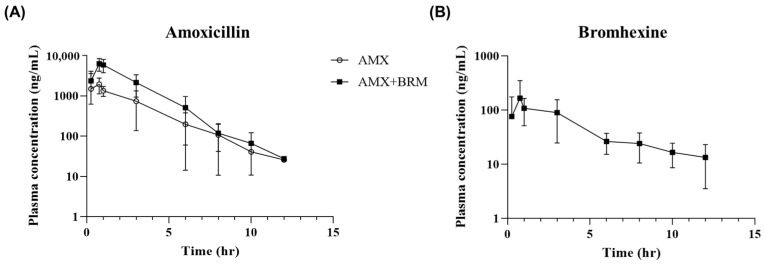
Curves showing the mean concentrations of (**A**) amoxicillin trihydrate (AMX) and (**B**) bromhexine hydrochloride (BRM) in plasma versus time in laying hens (n = 6/group) following the oral administration of AMX analytical standard at 50 mg/kg alone or in combination with 10 mg/kg BRM. The sars represent standard deviation (SD).

The plasma concentrations of AMX and BRM were analyzed following the oral administration of the analytical standards at 50 mg/kg AMX and 10 mg/kg BRM. AMX and BRM concentrations were detectable for up to 12 h following the last administration in both the AMX and AMX + BRM groups (Figure 1A,B). A comparison of the parameters between the AMX-only and AMX+BRM co-administration groups showed no differences in T_max_, T_1/2_, and k_el_ values, whereas the AUC_0–12h_ and C_max_ were significantly higher in the AMX+BRM co-administration group (Table 1). Meanwhile, although the assessment was incomplete due to the lack of oral bioavailability data for comparison between orally administered groups, the CL/F parameter was found to be significantly lower in the AMX+BRM group (3264 ± 1150 mL/h/kg) than the AMX group (9968 ± 2490 mL/h/kg). In addition, the V_d_/F value was significantly higher in the AMX+BRM group (6131 ± 2126 mL/kg) than the AMX alone group (17,568 ± 6176 mL/kg). The peak AMX concentrations in the plasma following oral administration were 2343 ± 1553 and 6816 ± 1677 ng/mL in the AMX and AMX+BRM groups, respectively (Table 1). The C_max_ for BRM was 208 ± 160 ng/mL and T_max_ was 1 (0.75~3) h (Table 2).

### 3.2. Egg Residue Analysis

The UPLC-MS/MS method was validated and found to be specific and linear (0~2 mg/kg) for AMX and BRM in eggs (R^2^ > 0.99). The LOD and LOQ of AMX were 0.679 and 2.239 µg/kg, while the corresponding values for BRM were 0.172 and 0.567 µg/kg, respectively. The recoveries of AMX and BRM in the plasma were 92.8 ± 5.2% and 88.9 ± 5.1%, respectively. All intra- and inter-day relative standard deviations for the plasma were < 7%.

**Figure 2:**
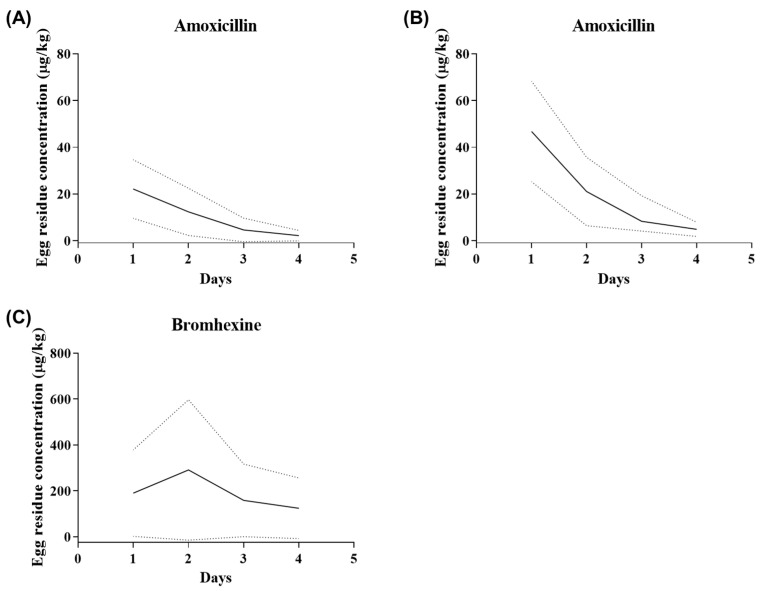
Curves showing the mean concentrations of (**A**,**B**) amoxicillin (AMX) and (**C**) bromhexine hydrochloride (BRM) in eggs of laying hens (n = 6/group) following the oral administration of AMX analytical standard at 50 mg/kg alone (**A**) or in combination with 10 mg/kg bromhexine (**B**,**C**). The solid lines represent the mean, while the dashed lines represent the 95% confidence intervals.

The egg residues of AMX and BRM were determined following the oral administration of 50 mg/kg AMX and 10 mg/kg BRM analytical standards (Figure 2A–C). The peak AMX concentrations in the egg, following oral administration, were 22.109 ± 5.066 and 46.768 ± 13.526 µg/kg in the AMX and AMX+BRM groups, respectively (Table 3). The BRM peak concentration in the egg after oral administration was 291.282 ± 123.407 µg/kg in AMX+BRM group (Table 4). The AMX and BRM contents of the eggs were reduced in a time-dependent manner. There was no decrease in egg production rate due to bromhexine administration.

## 4. Discussion

AMX is a β-lactam antibiotic commonly administered to laying hens in intensive farming systems [1,5]. Many studies have shown that different drug combinations, with various indications, can enhance the therapeutic effects of antibiotics. For example, the mucolytic agent BRM has been reported to influence the tissue distribution of AMX, resulting in superior clinical outcomes [12,17]. In addition, co-administration of AMX and BRM could enhance the efficacy of AMX through normalization of respiratory mucus and airway clearing [11]. In the present study, the effects of BRM co-administration on the pharmacokinetics of AMX in laying hens and the potential impact of egg residue were evaluated.

In the present study, BRM combination treatment enhanced the C_max_ and AUC values of AMX following oral gavage compared to AMX treatment alone. The V_d_/F was normalized to the oral bioavailability value from a previous study, which was approximately 63% [8]. When compared by applying a bioavailability of 63%, the V_d_/F of the AMX and AMX + BRM groups were 11,067 ± 3890 and 3862 ± 1339 mL/kg, respectively, indicating a significant decrease in the AMX + BRM group. However, it is reasonable to conclude that such a comparison is premature because the effect of BRM on the bioavailability (BA) of AMX cannot be confirmed without data from intravenous pharmacokinetic studies on AMX with and without BRM. However, in the present study, to evaluate the changes in antimicrobial efficacy due to BRM co-administration, parameters related to the efficacy of time-dependent antibiotics such as AMX, including T > MIC, were calculated under the assumption that the BA of AMX remained the same. Applying a MIC of 0.125 μg/mL [18], which has previously been reported as effective for Pasteurella multocida, one of the causative agents of respiratory disease, the BRM combination treatment showed a higher value (approximately 37%) than the AMX alone treatment (approximately 26%), with significant differences. Even when assuming a 100% oral absorption rate of AMX in the BRM co-administration group, the T > MIC difference was significantly higher in the BRM co-administration group. This result indicates that the co-administration of BRM could enhance the antimicrobial efficacy of AMX.

In the present study, AUC and C_max_ increased with BRM co-administration, whereas the T_1/2_, k_el_, T_max_, and CL/F values remained unchanged. However, without the exact values of AMX’s oral bioavailability of AMX in the presence or absence of BRM, it is difficult to accurately speculate how BRM affects either the absorption or excretion of AMX. Nevertheless, several reports have supported the enhanced systemic exposure of co-administered drugs under BRM treatment, reporting that co-administration of 1 mg/kg BRM hydrochloride with 20 mg/kg tilmicosin significantly increased the T_max_, C_max_, and AUC of tilmicosin, thereby accelerating the absorption of antibiotics in broiler chickens [19]. Further, Sumano et al. [20] demonstrated that BRM promoted the diffusion of furaltadone into bronchial secretions in broilers. Importantly, Taskar et al. [21] also reported that the co-administration of BRM increased the concentration of AMX in the sputum of patients.

Previous studies have similarly reported that mucolytic agents such as N-acetylcysteine (NAC), or surfactants, can enhance the intestinal absorption of water-soluble drugs by modulating the mucus of the intestinal mucosa [22]. The mechanisms underlying the action of BRM do not exactly align with those of NAC: NAC breaks disulfide bonds in mucoproteins, while BRM stimulates lysosomal enzyme activity to break down complex molecules in the mucus, thus reducing its viscosity. Nevertheless, it has been speculated that BRM may increase in vivo exposure to AMX through a similar mechanism. Reports recommending that BRM should not be administered to patients with gastric ulcers because of its potential to damage the stomach mucosal barrier partially support the evidence of BRM’s impact on the intestinal mucosa [23]. BRM also shows promise in respiratory applications; however, its specific effects on the intestinal mucosa require further investigation.

The mean egg concentration of AMX was significantly higher in the AMX + BRM group than in the AMX group; however, the withdrawal period calculated for AMX was 5 days in both groups. The Korean government’s Positive List System (PLS) set the MRL for AMX and BRM in eggs as 0.01 mg/kg [24]. In both groups, egg residues of AMX below the MRL (10 ppb) based on PLS were observed three days following the last oral administration. Previous studies have further reported that when 50 mg/kg AMX was orally administered to laying hens for consecutive 5 days, the withdrawal period was alternatively calculated as 9.11 days [7] and 6.5 days [25]. In the present study, BRM showed a relatively higher transfer rate into eggs, with residues persisting longer than AMX. The withdrawal period for BRM was set at 7 days, based on the PLS standards. According to the EMA, this drug should not be used in birds that produce eggs for human consumption before 4 weeks of the laying period [26]. The side effects of BRM include headache, dizziness, sweating, and allergic reactions, and drug residues in food can lead to adverse effects due to unintended drug administration, making it crucial to adhere to the withdrawal period of the drug for food safety. In this study, the results obtained under the condition of administering 10 mg/kg BRM orally along with AMX for three days indicated that the withdrawal period should be set at 7 days according to PLS standards. The present results obtained are based on the current settings, and compliance with existing regulations is essential to ensure the safe use of the drug.

AMX belongs to BCS class 1; it is a hydrophilic drug that is well absorbed orally, widely distributed, and excreted primarily in the urine, rather than through metabolism [27,28]. This drug is metabolized by oxidation, hydroxylation, and deamination processes, but is mainly excreted in the urine [29]. The hydrophilicity of AMX has been attributed to its short half-life, owing to the rapid elimination of the drug. BRM is a lipophilic drug belonging to BCS class 2, which is excreted in the urine and bile through extensive first-pass metabolism [30]. In the present study, although BRM was administered at a lower dose than AMX and showed lower plasma concentrations, it exhibited relatively higher residues in eggs compared to AMX. While discussions on the direct correlation between lipophilicity and residue patterns in eggs are ongoing, this is generally presumed to be due to the fact that lipophilic substances tend to distribute more readily into tissues compared to hydrophilic substances [31]. In the present study, both drugs were administered via oral gavage to laying hens; therefore, the results may differ from those when the drug is administered by mixing it with feed or drinking water in actual applications or farms.

## 5. Conclusion

The co-administration of AMX with BRM, which is known to help treat respiratory diseases, increased both the C_max_ and AUC of AMX. Although there was no significant difference in the egg withdrawal period for AMX between the AMX and BRM co-administered groups, BRM remained in eggs for longer than AMX. When drugs are co-administered, the withdrawal period should be set based on the drug with the longer residue time, and the egg residue patterns of such drugs must be considered conservatively to ensure food safety.

## Figures and Tables

**Table 1:** Pharmacokinetic profiles (mean ± standard deviation) of amoxicillin (AMX) single and in combination with bromhexine (BRM) in plasma of laying hens (n = 6) after oral gavage administration of AMX and BRM analytical standard at 50 mg/kg and 10 mg/kg, respectively.

Parameter	Unit	AMX	AMX+BRM
AUC_0–12h_	h*ng/mL	5178 ± 1344	16,856 ± 6229 *
AUC_0–inf_	h*ng/mL	5273 ± 1384	16,968 ± 6330 *
C_max_	ng/mL	2343 ± 1553	6816 ± 1677 *
T_1/2_	h	1.2 ± 0.1	1.3 ± 0.1
k_el_	/h	0.6 ± 0.1	0.5 ± 0.1
T_max_	h	0.75 (0.25~0.75)	0.875 (0.75~1)
V_d_/F	mL/kg	17,568 ± 6176	6131 ± 2126 *
CL/F	mL/h/kg	9968 ± 2490	3264 ± 1150 **

Values for T_max_ are presented as median (range). Other values are presented as mean ± standard deviation. * *p* < 0.05 and ** *p* < 0.01 vs. AMX group.

**Table 2:** Pharmacokinetic profiles (mean ± standard deviation) of bromhexine (BRM) in plasma of laying hens (n = 6) after oral administration of AMX and BRM analytical standard at 50 mg/kg and 10 mg/kg, respectively.

Parameter	Unit	BRM
AUC_0–12h_	h*ng/mL	582 ± 222
AUC_0–inf_	h*ng/mL	657 ± 249
C_max_	ng/mL	208 ± 160
T_1/2_	h	3.4 ± 0.7
k_el_	/h	0.22 ± 0.06
T_max_	h	1 (0.75~3)
V_d_/F	mL/kg	88,020 ± 56,198
CL/F	mL/h/kg	17,829 ± 9508

Values for T_max_ are presented as median (range). Other values are presented as mean ± standard deviation.

**Table 3:** Amoxicillin (AMX) residue concentration (mean, standard deviation (SD), upper) in eggs of laying hens (n = 6) after oral administration of 50 mg/kg AMX single and in combination with 10 mg/kg bromhexine (BRM).

Day		1	2	3	4
AMX	Mean (ppb)	22.109	12.350	4.556	<LOQ
	SD (ppb)	5.066	6.374	2.046	<LOQ
	Upper * (ppb)	88.527	66.296	31.978	13.831
AMX+BRM	Mean (ppb)	46.768	21.049	8.296	4.811
	SD (ppb)	13.526	9.219	4.388	2.458
	Upper * (ppb)	161.243	99.071	65.833	21.412

* Upper residue amount, 95% confidence interval with 99% upper tolerance limit.

**Table 4:** Bromhexine (BRM) residue concentration (mean, standard deviation (SD), upper) in eggs of laying hens (n = 6) after oral administration of amoxicillin (AMX) and BRM analytical standard at 50 mg/kg and 10 mg/kg, respectively.

Day		1	2	3	4
BRM	Mean (ppb)	190.475	291.282	159.133	124.516
	SD (ppb)	118.495	123.407	63.876	82.824
	Upper * (ppb)	1193.360	1909.360	996.658	825.498

* Upper residue amount, 95% confidence interval with 99% upper tolerance limit.

## Data Availability

The datasets used/analyzed during the current study are available from the corresponding author upon reasonable request.
